# Hypertriglyceridemic waist and subclinical atherosclerosis: a systematic review and meta-analysis

**DOI:** 10.3389/fcvm.2025.1660540

**Published:** 2026-01-05

**Authors:** Wei Xiong, Hua Fan, Yao You, Qingwen Yu, Ziyi Xin, Ting Tang, Xiyun Rao, Lanlan Feng, Yongmin Shi, Xuhan Tong, Jiake Tang, Xinyan Fu, Xingwei Zhang, Mingwei Wang, Hangzhou Luo

**Affiliations:** 1 Department of Science and Education, The First People's Hospital of Jiande, Hangzhou, China; 2 Office of Research and Innovation, The First Affiliated Hospital of Henan University of Science and Technology, Luoyang, China; 3 Department of Cardiology, Affiliated Hospital of Hangzhou Normal University, Hangzhou Normal University, Hangzhou, China; 4 Zhejiang Key Laboratory of Medical Epigenetics, Hangzhou Normal University, Hangzhou, China; 5 Engineering Research Center of Mobile Health Management System & Ministry of Education, Hangzhou Normal University, Hangzhou, China; 6 Hangzhou Institute of Cardiovascular Diseases, Hangzhou Normal University, Hangzhou, China; 7 Department of Cardiology, Hangzhou Lin'an Fourth People's Hospital, Hangzhou, Zhejiang, China

**Keywords:** subclinical atherosclerosis, hypertriglyceridemic waist, cardiovascular disease, carotid intima-Media thickness, meta-analysis

## Abstract

**Background:**

Atherosclerosis is a chronic, progressive inflammatory disease with a long asymptomatic period. Disease progression can eventually lead to acute cardiovascular events, such as myocardial infarction, unstable angina, and sudden cardiac death. Hypertriglyceridemic waist (HTGW) has received much attention recently as a cheap and non-invasive test. The purpose of our study was to explore whether the HTGW phenotype could be used as an effective test for subclinical atherosclerosis.

**Methods:**

We screened seven databases (PubMed, Cochrane Library, EMBASE, CNKI, WANFANG, CBM, and Web of Science) using both free terms and MeSH terms. Six published studies which evaluate the connection between HTGW and subclinical atherosclerosis were selected. Total carotid intima-media thickness (IMT) and carotid plaque are surrogate markers of subclinical atherosclerosis and predictors of cardiovascular events. Demographic and clinical data, data on HTGW and markers associated with subclinical atherosclerosis, along with odds ratios (OR), relative risks (RR), and hazard ratios (HR) with 95% confidence intervals were extracted. Data were then integrated using Stata 16.

**Results:**

A total of six studies conformed to inclusion criteria and revealed that HTGW is associated with subclinical atherosclerosis (OR = 2.39, 95% CI = 1.63–3.51).

**Conclusion:**

Available evidence supports the notion that HTGW can be an effective and inexpensive means of testing for atherosclerosis.

## 1 Introduction

Cardiovascular disease remains a leading cause of death in the United States, exceeding cancer and chronic lung disease (1). Coronary heart disease is the leading cause of all cardiovascular deaths, followed by stroke, hypertension and heart failure (1). Atherosclerosis is a chronic disease of the arterial wall and represents the underlying cause of the majority of cardiovascular events. Atherosclerosis precedes cardiovascular events and has a prolonged asymptomatic phase during which the course of the disease can be modified by lifestyle modifications and treatment (2). Therefore, early diagnosis of subclinical atherosclerosis has become a top priority. Early detection of subclinical atherosclerosis at this modifiable stage provides a critical window for implementing preventive strategies that can potentially avert progression to overt cardiovascular events, thereby reducing long-term morbidity and mortality (3).

Imaging technology has made rapid advances in the past few years, playing an integral role in medical research and clinical treatment. Invasive imaging methods include coronary angiography, intravascular ultrasound, and optical coherence tomography. Noninvasive imaging includes ultrasound, computed tomography, magnetic resonance imaging, and scintillation imaging. These methods can not only detect the structure and composition of atheromatous plaques, but also reflect the true thickness of the carotid wall by measuring the posterior (far) wall IMT of the carotid artery (4).

Carotid intima-media thickness (IMT) is a widely used surrogate marker for atherosclerosis worldwide. The carotid IMT can be simply, noninvasively, and reproducibly measured through B-mode carotid ultrasound. Carotid IMT is also a strong predictor of future cerebral and cardiovascular events (5, 6). The global prevalence of elevated carotid IMT, carotid plaques, and carotid stenosis in people aged 30–79 years in 2020 was 27.62%, 21.13%, and 1.5%, respectively (equivalent to 1,066.70 million, 815.75 million, and 57.79 million people, respectively). Carotid atherosclerosis is a substantial global burden, and its prevention and management require effective strategies (7).

The hypertriglyceridemic waist (HTGW) phenotype is characterized by a simultaneous increase in serum triglyceride (TG) and waist circumference (WC). One study has suggested that the simultaneous interpretation of waist circumference and fasting TG levels may help better identify individuals with concurrent hyperinsulinemia, high apolipoprotein B, and small, low-density lipoprotein phenotypes that are characterized by an increased risk of coronary heart disease (8). Subjects with HTGW tend to have an atherogenic lipid profile [significantly higher triglycerides, atherogenic index of plasma, non-high-density lipoprotein (HDL)-C, lower HDL-C and apoliprotein (Apo) A-1, and also higher total cholesterol and ApoB in women). The inclusion of HTGW as a simple, easily accessible, and inexpensive screening tool into daily clinical practice in primary care could lead to the detection of large numbers of subjects with cardiometabolic risk (9).

There is increasing interest in the relationship between high triglyceride phenotypes and cardiovascular disease (10). As such our aim was to explore whether HTGW is effective as a non-invasive test to predict early atherosclerosis.

## 2 Materials and methods

### 2.1 Search strategy

According to the instructions of the PRISMA statement, we performed a systematic literature search of seven databases (PubMed, Cochrane Library, EMBASE, CNKI, WANFANG, CBM, and Web of Science) from the inception dates to June 11, 2025. Free terms and MeSH terms, such as [(subclinical atherosclerosis) OR (coronary calcium, subclinical atherosclerosis) OR (preclinical atherosclerosis)] AND ((“Hypertriglyceridemic waist” [MeSH]) OR (HTGW phenotype) OR (Waist, Hypertriglyceridemic) OR (HTGW) OR (Enlarged Waist Elevated Triglycerides)) were used to find relevant articles. The references of articles identified through searching these terms were also examined to find any suitable study.

### 2.2 Inclusion and exclusion criteria

The inclusion criteria were as follows: (1) prospective studies, cross-sectional studies, and retrospective analyses; (2) original research revealing the relationship between HTGW and subclinical atherosclerosis; and (3) odds ratios (ORs) offered or at least able to be calculated from the provided data;

The exclusion criteria were as follows: (1) only triglycerides *or* waist circumference increased in the experimental group, but not both; (2) lack of healthy control groups; (3) animal experiments, review literatures, or case reports; (4) duplicate papers; (5) lack of important primary data.

### 2.3 Data extraction

Two researchers (You Yao and Wei Xiong) each independently screened the title and abstract of each article returned from our search query and examined the associated paper for suitability for inclusion in our study. From those papers selected for inclusion in the study, we extracted the following characteristics into an electronic database: (1) study: first author, publication year, and study design; (2) participants: subject number, age of the HTGW and control groups; and (3) assessment methods.

### 2.4 Quality assessment of included studies

1. Quality assessment of case-control and cohort studies

Among those studies we selected for inclusion were one case-control and one cohort study. We employed the Newcastle-Ottawa Scale (NOS) to assess the quality of each study. The case-control study (11) scored five points on this scale, whereas the cohort study (12) scored eight points.

1. Quality assessment of four cross-sectional studies (13–16)

We used the JBI Critical Appraisal Checklist for analytical cross-sectional studies to assess the quality of each cross-sectional study.

A detailed summary of the quality assessment for each study, including scores for all individual criteria, is presented in Supplementary Table S1.

### 2.5 Statistical analysis

We performed all statistical analyses using Stata 16. The association between HTGW and subclinical atherosclerosis was expressed as odds ratios (ORs) with corresponding 95% confidence intervals (CIs). A random-effects model was applied to synthesize the results, using the DerSimonian–Laird estimator for the between-study variance. The 95% CIs for the pooled effect were calculated using the standard Wald-type method, and the Hartung–Knapp adjustment was not applied.

Between-study heterogeneity was assessed using Cochran's *Q* test and quantified using the *I*^2^ statistic and *τ*^2^. A 95% prediction interval (PI) was also calculated to estimate the range of true effects in future studies. Assessment for publication bias was omitted due to the small number of included studies, as such methods are considered unreliable with fewer than 10 studies.

## 3 Results

### 3.1 Search results

**Figure 1:**
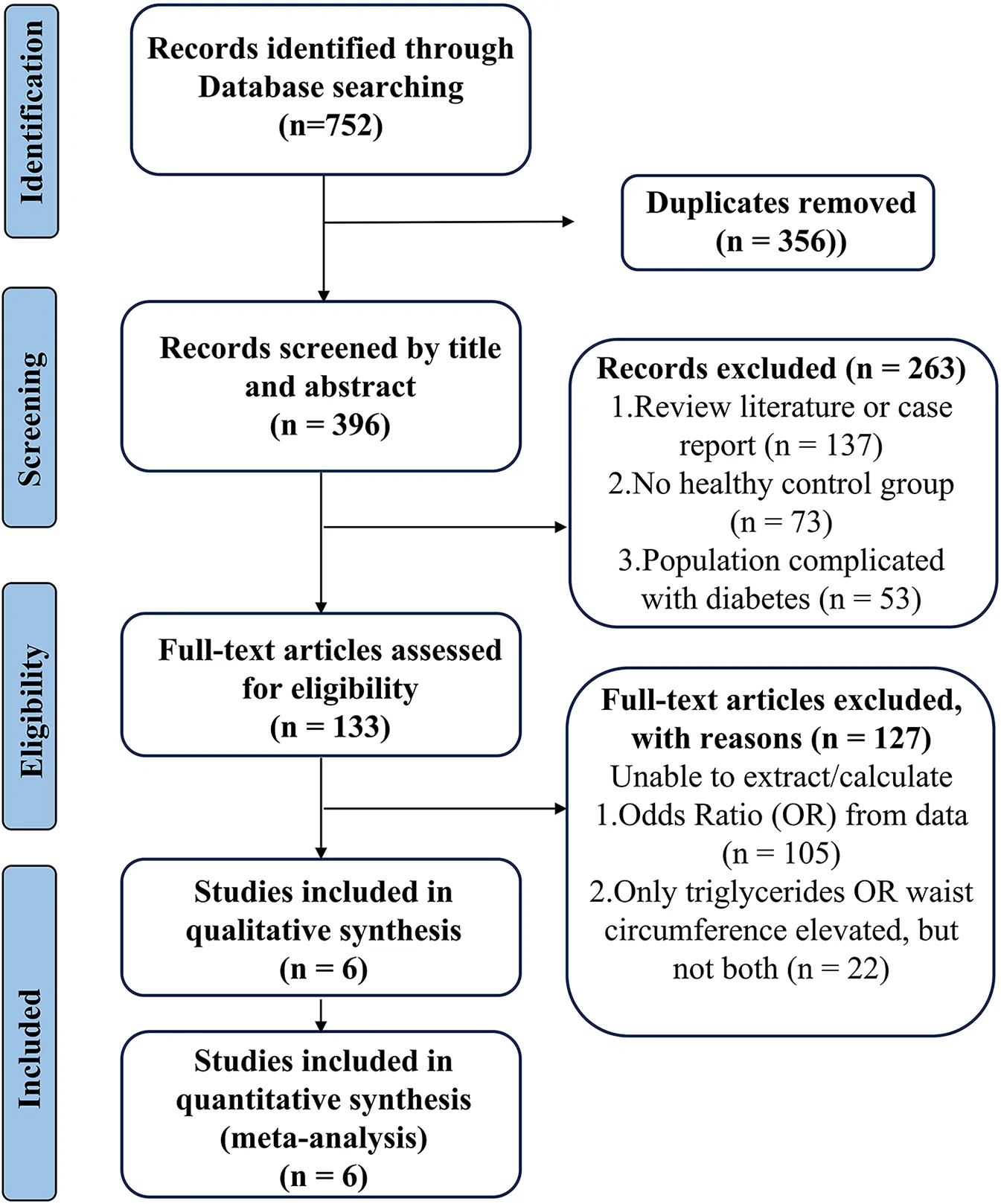
A flow chart showed the study selection process according to the PRISMA statement.

The process of screening and selecting papers is shown (Figure 1). We retrieved a total of 752 potentially related papers by electronic and manual search. After removing duplicates and screening the title and abstract of each paper, we found 133 related papers. After reviewing the full articles, 127 articles were further excluded due to (1) lack of OR within the original data or (2) only triglycerides or waist circumference being increased in the experimental group. Finally, six unique observational studies were identified as eligible for inclusion in the meta-analysis.

### 3.2 Characteristics of the included studies

At last, our dataset consisted of a total of six studies comprising a total of 2,136 participants, which included one cohort study, one case-control study, and four cross-sectional studies. Three of them assessed subclinical atherosclerosis using IMT (11, 14, 15), two of them evaluated plaque presence (12, 16), and one study used carotid artery ultrasonography (13).

### 3.3 Meta-analysis

**Figure 2:**
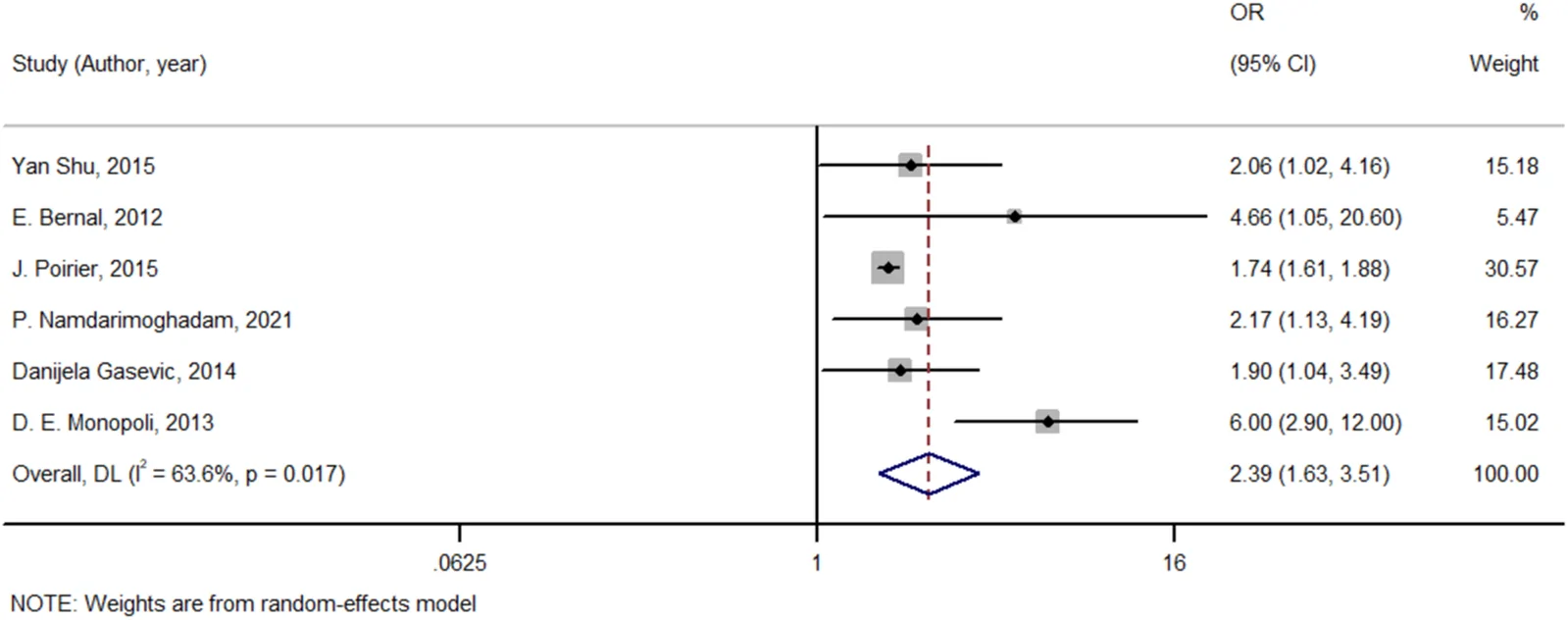
Forest plot presents subclinical atherosclerosis prevalence in HTGW subjects and controls.

An association between HTGW and subclinical atherosclerosis was identified in six studies (Figure 2). The summary OR was 2.39 (95% CI = 1.63–3.51), indicating a significant association. We observed substantial between-study heterogeneity, as indicated by the Cochran's *Q* test (*Q* = 13.75, *p* = 0.0173) and the *I*^2^ statistic (*I*^2^ = 63.6%). The estimated between-study variance was *τ*^2^ = 0.1238. The 95% prediction interval was wide [0.85–6.72].

**Figure 3:**
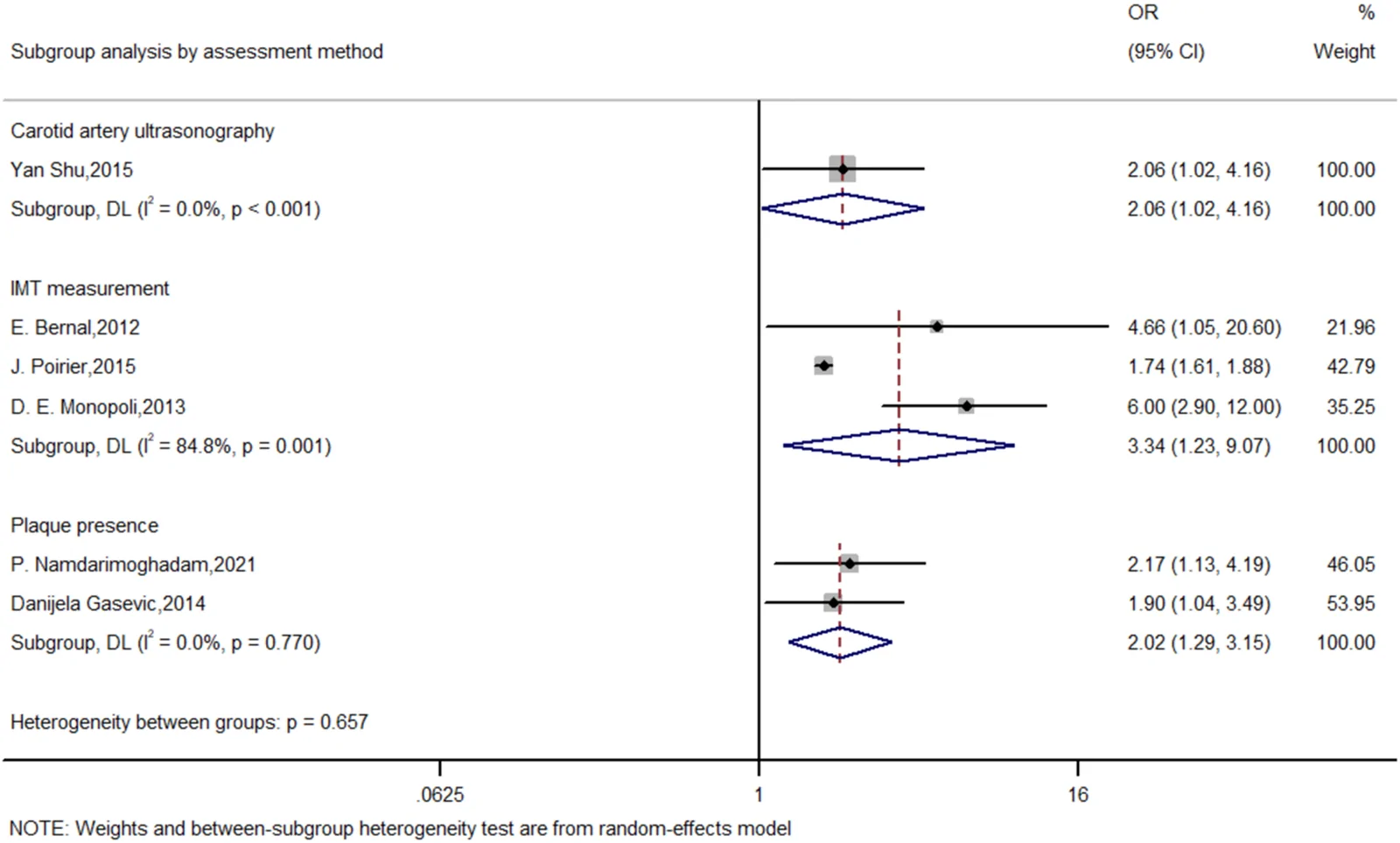
Forest plot of subgroup analysis for the association between the HTGW phenotype and subclinical atherosclerosis, stratified by assessment method.

The I² statistic revealed substantial heterogeneity among the included studies (I² = 63.6%, *p* = 0.017). To explore the sources of this heterogeneity, we performed a subgroup analysis stratified by the method used to assess subclinical atherosclerosis (Figure 3). The association remained significant across all diagnostic methods: the pooled OR was 2.06 (95% CI: 1.02–4.16) for the subgroup defined by general carotid ultrasonography, 3.34 (95% CI: 1.23–9.07) for studies using IMT measurement, and 2.02 (95% CI: 1.29–3.15) for those based on plaque presence. Notably, the heterogeneity was substantial within the IMT measurement subgroup (I² = 84.8%, *p* = 0.001), whereas no heterogeneity was observed in the plaque presence subgroup (I² = 0.0%, *p* = 0.770). The test for differences between these subgroups was not statistically significant (*p* = 0.657).

### 3.4 Sensitivity analysis

**Figure 4:**
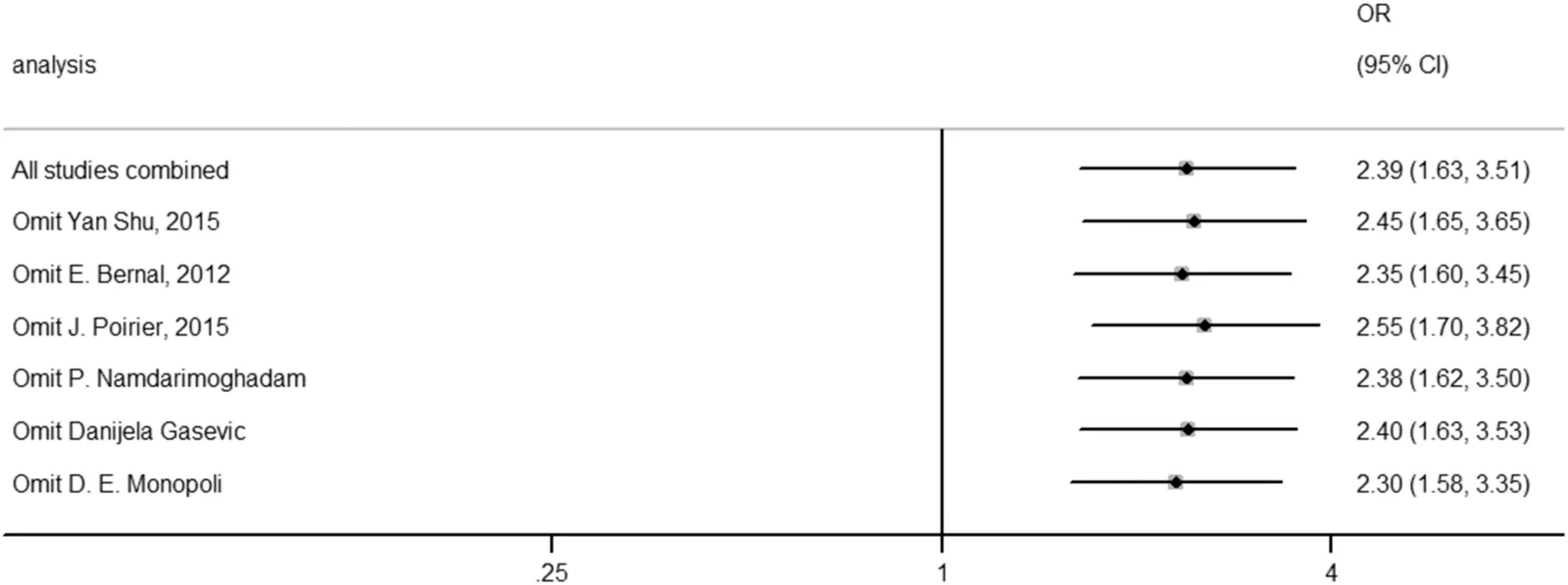
Leave-one-out sensitivity analysis of the association between the hypertriglyceridemic waist phenotype and subclinical atherosclerosis.

We performed a leave-one-out sensitivity analysis to assess result stability. The association remained statistically significant after sequentially excluding each study, with odds ratios ranging from 2.30 (95% CI: 1.58–3.35) to 2.55 (95% CI: 1.70–3.82) (Figure 4).

## 4 Discussion

This systematic review shows a connection between HTGW and subclinical atherosclerosis based on six high-quality studies involving a total of 2,136 subjects. Our meta-analysis demonstrated that individuals with the HTGW phenotype had significantly higher odds of subclinical atherosclerosis (OR = 2.39, 95% CI = 1.63–3.51). We thus conclude that HTGW may serve as a potential indicator for subclinical atherosclerosis.

In recent years, with the development of imaging techniques, it has become possible to detect and monitor subclinical atherosclerosis at an early stage. Among them, non-invasive imaging plays an important role in the identification, stratification, and optimal management of subjects with early atherosclerosis (17). Noninvasive imaging methods are superior to invasive techniques because they are safer, especially in asymptomatic populations (18, 19). B-mode ultrasound of carotid intima-media thickness (IMT) and the presence of plaque has long been used as a surrogate measure of subclinical disease (20, 21). Furthermore, arm/peripheral flow-mediated dilation, arterial stiffness, and coronary calcium scores are strong indicators of subclinical atherosclerosis (22). Despite the continuous improvement and innovation of imaging technology, however, there remains a need to find a simpler and more convenient screening method for large-scale screening and detection of subclinical atherosclerosis in order to guide clinical and healthcare management.

The HTGW phenotype, a combination of increased waist circumference and elevated fasting triglyceride concentration, can be used as a screening phenotype to identify atherogenic potential (23). Its clinical utility lies in the synergistic value as a simple composite indicator, which may help identify high-risk metabolic profiles more effectively than evaluating triglycerides or waist circumference in isolation. A European cohort study showed that HTGW was associated with worse cardiometabolic risk profile and an increased risk of coronary artery disease (24). It was showed that the HTGW phenotype was independently associated with the risk of cardiovascular disease in China. The HTGW phenotype may thus be a simple but useful tool for screening individuals at high risk for future cardiovascular disease and holds particular promise for initial risk stratification in resource-limited settings (25). However, further comparative studies against established risk assessment tools are needed to fully demonstrate its added clinical benefit. The correlation between HTGW and atherosclerosis was expressed in different age groups (26, 27). Our results support HTGW phenotype as an indicator of early subclinical atherosclerosis screening. Therefore, HTGW phenotype shows promise as a screening candidate, though its clinical utility requires validation through prospective studies. When interpreting these findings within the broader context of cardiovascular risk assessment, the HTGW phenotype should be viewed as a complementary tool rather than a replacement for established risk scores. Its value lies in identifying individuals who might be misclassified as low risk by traditional factors alone, particularly those with metabolic dysfunction not fully captured by conventional lipid profiles or anthropometric measures (8, 28). This synergistic approach could enhance early detection of at-risk populations while maintaining cost-effectiveness in resource-constrained settings.

The study covers clinical research on HTGW and subclinical atherosclerosis over the past 15 years. This is the first meta-analysis to reveal the predictive effect of HTGW phenotype on subclinical atherosclerosis. We further demonstrated the stability of our results through subgroup analyses based on different assessment methods of subclinical atherosclerosis. Overall, our results are consistent with previous experimental and clinical evidence supporting the HTGW phenotype as an indicator of screening for early subclinical atherosclerosis.

However, there are still some significant limitations concerning this meta-analysis. First, the evidence base remains limited, with only six studies comprising 2,136 participants, which restricts the statistical power and generalizability of our findings. This small number of studies also precluded a formal assessment of publication bias, which is considered unreliable with fewer than 10 studies. The included studies exhibited considerable methodological diversity, encompassing cross-sectional, case-control, and cohort designs with variable quality scores (including one case-control study that scored only 5 out of 9 points on the Newcastle-Ottawa Scale); consequently, significant statistical heterogeneity was observed (*I*^2^ = 63.6%). This heterogeneity stems from several factors: firstly, the studies employed different inclusion and exclusion criteria, particularly variations in diagnostic cutoffs for triglycerides and waist circumference. Secondly, the patient populations differed, with most having various concomitant cardiovascular risk factors and disease activity statuses. Additionally, the observational nature of the included studies, which are primarily cross-sectional, limits our ability to establish a causal relationship between HTGW and subclinical atherosclerosis. However, our ability to probe these sources was limited, as subgroup analyses by study design or population characteristics were not feasible. Notably, a subgroup analysis by assessment method revealed that the heterogeneity was primarily driven by studies using IMT measurement (*I*^2^ = 84.8%), which represents very high heterogeneity and substantially limits the reliability of conclusions drawn from this particular subgroup. While those based on plaque presence showed no heterogeneity (*I*^2^ = 0.0%). Nevertheless, the test for between-subgroup differences was not significant, and moreover, a consistent direction and statistical significance of the association was maintained across all subgroups and HTGW definitions. Therefore, although methodological variability and residual heterogeneity limit the strength of causal inference, the robustness and consistency of the pooled effect (OR = 2.39, 95% CI = 1.63–3.51) across diverse study designs support a association between HTGW and subclinical atherosclerosis. Sensitivity and subgroup analyses further confirmed the stability of this association, indicating that no single study disproportionately influenced the overall result. Nonetheless, the precise source of heterogeneity—particularly within the IMT subgroup—remains uncertain and should be explicitly acknowledged as a major limitation. Future large-scale prospective studies with standardized diagnostic criteria and measurement protocols are warranted to validate these findings and minimize residual heterogeneity.

In conclusion, despite the methodological limitations and substantial heterogeneity discussed above, the HTGW phenotype was significantly associated with subclinical atherosclerosis in our meta-analysis. The pooled odds ratio of 2.39 indicates that the odds of subclinical atherosclerosis were approximately 2.4 times higher in individuals with the HTGW phenotype. Therefore, in order to provide timely prevention and health management programs, we need to pay more attention to patients with high triglyceride levels and waist circumference in the clinic.

## Data Availability

The original contributions presented in the study are included in the article/Supplementary Material, further inquiries can be directed to the corresponding authors.
