# Supplementary material for: Hypertriglyceridemic waist and subclinical atherosclerosis: a systematic review and meta-analysis
Source: Front Cardiovasc Med. 2026 Jan 5;12:1660540. doi: 10.3389/fcvm.2025.1660540 (PMC12812524; doi:10.3389/fcvm.2025.1660540)
Supplement: Supplementary file 1 [file Table1.docx]

**Supplementary Table 1. Detailed Quality Assessment of Included Studies**

| **Study (Author, Year)** | **Study Design** | **Tool Used** | **Overall Score** | **Key Assessment Domains and Scores** |
| --- | --- | --- | --- | --- |
| Monopoli, D. E. et al. (2013) | Case-Control | NOS | 5/9 | **Selection:** ★★★☆ **Comparability:** ★★☆☆ **Exposure:** ★★☆☆ |
| Namdarimoghaddam, P. et al. (2021) | Cohort | NOS | 8/9 | **Selection:** ★★★★ **Comparability:** ★★☆☆ **Outcome:** ★★★☆ |
| Shu, Y. et al. (2015) | Cross-Sectional | JBI | Satisfactory* | **Participants:** Yes **Criteria:** Yes **Exposure:** Yes **Outcome:** Yes **Confounding:** Yes **Strategies:** Yes |
| Bernal, E. et al. (2012) | Cross-Sectional | JBI | Satisfactory* | **Participants:** Yes **Criteria:** Yes **Exposure:** Yes **Outcome:** Yes **Confounding:** Yes **Strategies:** Yes |
| Poirier, J. et al. (2015) | Cross-Sectional | JBI | Satisfactory* | **Participants:** Yes **Criteria:** Yes **Exposure:** Yes **Outcome:** Yes **Confounding:** Yes **Strategies:** Unclear |
| Gasevic, D. et al. (2014) | Cross-Sectional | JBI | Satisfactory* | **Participants:** Yes **Criteria:** Yes **Exposure:** Yes **Outcome:** Yes **Confounding:** Yes **Strategies:** Yes |

**Abbreviations:** NOS, Newcastle-Ottawa Scale; JBI, Joanna Briggs Institute Critical Appraisal Checklist.
* The JBI checklist does not provide a numeric summary score but assesses whether specific methodological criteria are met. "Satisfactory" indicates that the study met the majority of the key criteria. The detailed, itemized ratings for each study are provided in the supplementary material.
